# Risk and Resilience Factors During the COVID-19 Pandemic: A Snapshot of the Experiences of Canadian Workers Early on in the Crisis

**DOI:** 10.3389/fpsyg.2020.580702

**Published:** 2020-12-03

**Authors:** Simon Coulombe, Tyler Pacheco, Emily Cox, Christine Khalil, Marina M. Doucerain, Emilie Auger, Sophie Meunier

**Affiliations:** ^1^Department of Industrial Relations, Université Laval, Quebec City, QC, Canada; ^2^Department of Psychology, Wilfrid Laurier University, Waterloo, ON, Canada; ^3^Department of Psychology, Université du Québec à Montréal, Montreal, QC, Canada

**Keywords:** COVID-19, mental health, workers, wellbeing, stressors, resilience, ecological model

## Abstract

Research highlights several risk and resilience factors at multiple ecological levels that influence individuals’ mental health and wellbeing in their everyday lives and, more specifically, in disaster or outbreak situations. However, there is limited research on the role of these factors in the early days of the COVID-19 crisis. The present study examined if and how potential risk factors (i.e., reduction in income, job insecurity, feelings of vulnerability to contracting the virus, lack of confidence in avoiding COVID-19, compliance with preventative policies) and resilience factors (i.e., trait resilience, family functioning, social support, social participation, and trust in healthcare institutions) are associated with mental health and well-being outcomes, and whether these resilience factors buffer (i.e., moderate) the associations between risk factors and said outcomes. One to two weeks after the government recommended preventative measures, 1,122 Canadian workers completed an online questionnaire, including multiple wellbeing outcome scales in addition to measures of potential risk and resilience factors. Structural equation models were tested, highlighting that overall, the considered risk factors were associated with poorer wellbeing outcomes, except social distancing which was associated with lower levels of stress. Each of the potential resilience factors was found to have a main effect on one or more of the wellbeing outcomes. Moderation analysis indicated that in general these resilience factors did not, however, buffer the risk factors. The findings confirm that the COVID-19 crisis encompasses several stressors related to the virus as well as to its impact on one’s social, occupational, and financial situation, which put people at risk for lower wellbeing as early as one to two weeks after the crisis began. While several resilience factors emerged as positively related to wellbeing, such factors may not be enough, or sufficiently activated at that time, to buffer the effects of the numerous life changes required by COVID-19. From an ecological perspective, while mental health professionals and public health decision-makers should offer/design services directly focused on mental health and wellbeing, it is important they go beyond celebrating individuals’ inner potential for resilience, and also support individuals in activating their environmental resources during a pandemic.

## Introduction

The COVID-19 crisis has had, and continues to have, a serious impact on individuals throughout the world (Brooks et al., 2020; Xiao et al., 2020). As a result of the pandemic, individuals are facing continuous changes in various aspects of their lives, such as health, employment, and family life (Gangopadhyaya and Garrett, 2020; Xiao et al., 2020). This accumulation of multiple sources of stress could increase feelings of psychological distress and decrease feelings of wellbeing for many individuals.

Wellbeing can be defined as the evaluation, either positive or negative, of one’s life and quality of functioning in life (Magyar and Keyes, 2019). This definition is in accordance with second wave positive psychology, which posits that wellbeing should be understood based on the situational context in which individuals may experience a mix of positive (e.g., positive affect) and negative (e.g., distress) wellbeing (Wong, 2011; Lomas and Ivtzan, 2016). Further, wellbeing can include both hedonic (e.g., low levels of stress) and eudaimonic (e.g., meaning in life) aspects (Magyar and Keyes, 2019). As an important, yet still understudied component of wellbeing, meaning in life refers to “the extent to which people comprehend, make sense of, or see significance in their lives, accompanied by the degree to which they perceive themselves to have a purpose, mission, or overarching aim in life” (Steger et al., 2009, p. 43).

**FIGURE 1:**
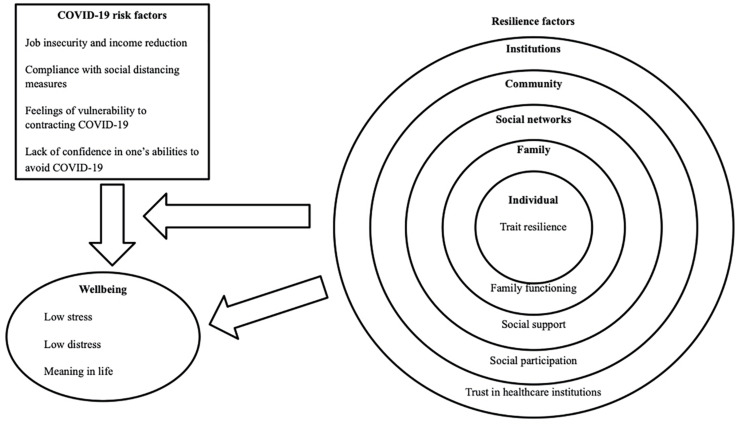
Graphical representation of the effects of potential risk factors on wellbeing and direct and moderating effects of potential resilience factors at multiple ecological levels.

Considering both hedonic and eudaimonic, and positive and negative indicators of wellbeing, the present research will explore if potential risk and resilience factors are associated with the mental health and wellbeing outcomes of Canadian workers during the first two weeks after COVID-19 preventative policies were instituted and, further, whether these resilience factors act as buffers (i.e., moderators) against the negative impacts of the identified risk factors. Previous research indicates that higher levels of fear or distress within the initial time period after a traumatic event or crisis can predict future psychological maladjustment (Udwin et al., 2000; La Greca et al., 2013). For instance, research conducted following the 9/11 attacks showed that positive and negative emotions experienced after the tragedy predicted long-term development of depression, resilience, and post-traumatic growth (Fredrickson et al., 2003). Thus, it is important to explore the risk and resilience factors impacting individuals in the first weeks of the COVID-19 crisis, which may provide recommendations on how to better help these individuals thrive during and after the crisis. Figure 1 presents a graphical representation of the considered risk and resilience factors, which will be described in the next sections. When we were able to retrieve studies that have specifically established the directionality or the causality of the relationships between these factors and wellbeing, this will be mentioned. When such studies are not explicitly mentioned, we mostly use the terms “relationship” or “association” to refer to the general relation between these factors and wellbeing. However, from a conceptual perspective focused on sources of risk and resilience, we conceptualize the identified risk and resilience factors as impacting wellbeing.

### Risk Factors

Previous research highlights several risk factors that can negatively impact an individuals’ mental health and wellbeing in situations of adversity, including job insecurity or job loss (Virtanen et al., 2005; Harvey et al., 2017; Lorenz et al., 2018), and financial hardships (Lorenz et al., 2018; Gangopadhyaya and Garrett, 2020). However, previous research on the impact of risk factors during a global pandemic, such as the COVID-19 crisis, is limited. Further, during a pandemic situation, individuals may also experience pandemic-specific factors that negatively impact their mental health and wellbeing, including feelings of vulnerability to contracting the COVID-19 virus, and compliance with preventative policies and recommendations (i.e., social distancing).

#### Job Insecurity and Income Reduction

Previous literature has identified several work-related factors, such as job insecurity, low job control, high psychological demands, and low social support, as having a negative impact on an employee’s mental health and wellbeing (Virtanen et al., 2005; Nieuwenhuijsen et al., 2010; Schmidt et al., 2014; Harvey et al., 2017). Specifically, these factors were associated with increased risk of depression, anxiety, and other stress-related disorders, such as adjustment disorders (Virtanen et al., 2005; Nieuwenhuijsen et al., 2010; Schmidt et al., 2014; Harvey et al., 2017).

Although current research in the context of the COVID-19 crisis is limited, findings from previous research not conducted in the context of a public health crisis may provide some guidance for the present study. For example, factors related to occupational uncertainty (e.g., control over one’s job, job insecurity, job loss) were associated with increased risk and severity of mental health symptoms in previous research. In a study by Lorenz et al. (2018), approximately half of the 303 participants experienced medium to high severity of adjustment disorder symptoms upon losing their job. While studies assessing the potential causal effect of job loss on wellbeing have yielded mixed findings (Kuhn et al., 2009; Salm, 2009; Schmitz, 2011), there is some evidence that job loss has a causal effect on mental wellbeing, but not physical health (Kuhn et al., 2009). These findings are in accordance with those from a systematic review of the literature, which found that temporary employment status was associated with higher levels of psychological morbidity (e.g., psychological distress, depression, fatigue) compared to individuals who possess permanent employment (Virtanen et al., 2005). In addition, one study found that job insecurity was associated with poor wellbeing and an increase in psychosomatic and physical complaints (Witte, 1999). Further, job insecurity has also been found to negatively influence job performance through a reduction in subjective wellbeing (Darvishmotevali and Ali, 2020). Examination of cross-lagged effects suggests that it is indeed job insecurity that predicts later mental health issues, and not the reverse (Hellgren and Sverke, 2003). However, these studies were not conducted during a pandemic situation; it is possible that occupational uncertainty will have an even greater impact on the mental health and wellbeing of individuals experiencing it in the context of the COVID-19 crisis, in which people are experiencing additional and novel stressors.

In addition to these findings, in a non-pandemic context, Lorenz et al. (2018) found that many participants also reported having additional financial life stressors associated with job loss, with approximately one third of individuals experiencing financial problems. These findings are particularly relevant to the current COVID-19 crisis as many individuals are experiencing financial issues, such as changes to, or loss of, income (Coibion et al., 2020; Gangopadhyaya and Garrett, 2020). In a study by Mihashi et al. (2009), it was found that income reduction caused by quarantine measures during the severe acute respiratory syndrome (SARS) outbreak was associated with psychological disorders (i.e., as measured by the General Health Questionnaire) for approximately one quarter of participants. Thus, as individuals are experiencing occupational uncertainty and financial issues at a higher than usual rate (Government of Canada Statistics, 2020) due to the COVID-19 crisis, it is important to further explore how this situation has impacted employee’s mental health and wellbeing (Coibion et al., 2020; Gangopadhyaya and Garrett, 2020).

#### Feelings of Vulnerability to Contracting COVID-19

In addition to employment and financial stressors, many individuals are likely experiencing new risk factors to their mental health and wellbeing that are associated specifically with the pandemic situation. In particular, previous research about other outbreak situations identifies fear of infection as a common risk factor impacting individuals’ mental health and wellbeing (Maunder et al., 2003; Reynolds et al., 2008; Desclaux et al., 2017). During the Ebola outbreak, individuals living in Senegal reported feeling particularly vigilant about any physical symptoms they experienced, for fear of contracting the virus (Desclaux et al., 2017). As a result, several participants reported anxiety-induced insomnia, demonstrating that constant vigilance and feelings of vulnerability may have a negative impact on one’s mental health and wellbeing.

These findings are similar to those demonstrated by Maunder et al. (2003) in which healthcare staff caring for patients with SARS experienced feelings of anxiety about contracting the disease. In addition to feelings of personal vulnerability to infection, participants within previous studies have also indicated feelings of fear and guilt about infecting others including family members, friends, and the healthcare workers caring for them during an outbreak situation (Maunder et al., 2003). For instance, in a study conducted in Taiwan during the SARS outbreak, healthcare participants indicated feelings of fear about infecting their family members (Bai et al., 2004). As a result, “52 staff members (15 percent) did not go home after work during the outbreak” (Bai et al., 2004, p. 1057). Overall, such experiences have been found to be associated with low mood, poor quality of sleep, irritability, in addition to other mental and physical health issues (Hawryluck et al., 2004; Reynolds et al., 2008; Brooks et al., 2020). As previous research has focused primarily on the experiences of vulnerability among healthcare providers, it is important to understand how a large-scale global pandemic influences feelings of vulnerability to contracting the virus and, further, how these feelings impact the mental health and wellbeing of the general population of workers.

#### Compliance With Social Distancing Measures and Lack of Confidence in One’s Abilities to Avoid COVID-19

Another risk factor that may be particularly relevant during the current global pandemic is compliance with preventative policies, such as social distancing and quarantining. In a review of the literature on the psychological impacts of quarantine, it was found that individuals who had been quarantined were more likely to report high rates of mental health symptoms (e.g., psychological distress, depression, anxiety, post-traumatic stress symptoms) due to enhanced feelings of isolation and distance from the outside world (Hawryluck et al., 2004; Abel and McQueen, 2020; Brooks et al., 2020). For example, two studies conducted in Canada during the SARS outbreak demonstrated that longer duration of quarantine and compliance with preventative measures was associated with increased psychological distress and more symptoms of post-traumatic stress disorder (Hawryluck et al., 2004; Reynolds et al., 2008). In a recent editorial, Abel and McQueen (2020) suggest that social distancing may contribute to worse mental health issues, especially for those from collectivist cultures, in which social connections are valued more deeply.

Mental health symptoms have also been associated with other stressors related to one’s lack of confidence in their ability to prevent contracting the virus, such as frustration with having inadequate information and supplies, and feelings of low self-efficacy in controlling the outbreak. For example, Hawryluck et al. (2004) found that many individuals did not feel adequately informed about how SARS was transmitted and how it could be controlled (e.g., disinfection of personal items), which induced feelings of anxiety and anger among participants. In addition to these findings, Mækelæ et al. (2020) found that less distress was associated with greater feelings of control of the COVID-19 outbreak and the perception that one’s actions were efficacious among participants from six countries (e.g., Brazil, Colombia, Germany, Israel, Norway, United States). These findings suggest that lack of confidence in one’s abilities with regards to avoiding the virus may be a risk factor for individuals’ mental health and wellbeing during a global pandemic situation such as the COVID-19 crisis.

### Resilience Factors

While the current situation is likely to increase risk factors such as those outlined above, among others, individuals also tend to show considerable resilience in difficult situations, which can act as a buffer against the negative impacts of stressors (Lee et al., 2013; Dickinson and Adams, 2014). Previous research has explored resilience from both an individual perspective (i.e., one’s ability to bounce back) as well as from a socio-ecological perspective (i.e., “the process of biological, psychological, social, and ecological systems interacting in ways that help individuals to regain, sustain, or improve their mental wellbeing” in the face of risk factors, Ungar and Theron, 2020, p. 441). While one’s level of personal resilience abilities and skills (i.e., ‘trait resilience’) may be a protective factor, previous research from a variety of fields such as psychology, architecture, and human ecology has demonstrated the importance of considering not only an individual’s inner strengths, but also their social environments and the availability of culturally relevant resources within them (Ungar and Theron, 2020). In particular, the previous literature has identified several socio-ecological factors for resilience, such as family functioning (Tam et al., 2004; Rabelo et al., 2016), social support (Tam et al., 2004; Xiao et al., 2020), social participation (Kaplan et al., 2012; Ding et al., 2015) and trust in healthcare institutions (Ahnquist et al., 2010; Ward, 2017) that can play a role in maintaining people’s mental health and wellbeing. However, it is unclear if and how these potential protective factors buffer the impacts of risk factors during the current COVID-19 crisis.

#### Trait Resilience

Previous research demonstrates that resilience can be conceptualized as a personal trait or state in which individuals are able to adapt to or overcome adversity (Lee et al., 2013). For example, in a study conducted with North Korean refugees living in South Korea, it was found that the relationship between family cohesion and depression was fully mediated by trait resilience (Nam et al., 2016). In particular, trait resilience was not only significantly correlated with depression, but also decreased the power of family cohesion in predicting depression from −0.41 to −0.19. Upon conducting a logistical regression, the association between the independent and dependent variable was nullified once trait resilience was controlled for Nam et al. (2016). In accordance with these findings, a meta-analysis demonstrated that “trait resilience was negatively correlated with negative indicators of mental health and positively correlated with positive indicators of mental health” (Hu et al., 2015, p. 24). While resilience trait is often considered to be an antecedent of wellbeing, based on a study conducted with college students in China (Wu et al., 2020), it is possible that resilience plays a causal role in wellbeing, which, in turn, plays a causal role in subsequent levels of resilience.

In addition to these findings in contexts other than the current crisis, Kavčič et al. (2020) conducted a study on the role of trait resilience in one’s psychological functioning during the current COVID-19 crisis in which resilience was found to be positively associated with Slovene adults’ mental health and perceived stress. As the current COVID-19 crisis has raised many challenges and uncertainties within the lives of individuals across the globe, it is important to further explore the role of trait resilience as a protective factor for one’s mental health and wellbeing.

#### Family Functioning

Previous research demonstrates that, while poor family functioning can amplify mental health issues and symptoms, positive family functioning can act as a protective factor against the impacts of stressors on mental health. For example, several studies have found that exposure to a family member who had contracted Ebola was associated with low family wellbeing, increased family conflict, and exclusion or rejection from family members (Rabelo et al., 2016; Green et al., 2018). Consequently, those who survived Ebola reported experiencing stigma and isolation. Alternatively, a systematic review of the literature found that support from one’s family was associated with reduced risk of mental health issues and symptoms among healthcare workers during the SARS outbreak (e.g., anxiety, Brooks et al., 2018). In addition to these findings, a study with North Korean refugees found that family cohesion was associated with lower levels of depression (Nam et al., 2016). Thus, as family functioning may act as a protective factor, it is important to understand the role of family functioning in mental health and wellbeing experiences during the current COVID-19 crisis.

#### Social Support

In addition to family functioning, social support has also been found to be an associated with better mental health and wellbeing during outbreak situations throughout the world (Tam et al., 2004; Pan et al., 2005; Rabelo et al., 2016; Xiao et al., 2020). For example, survivors of Ebola indicated that support from friends and family members was an effective coping strategy for managing mental distress (Rabelo et al., 2016). Similarly, in a study by Pan et al. (2005), researchers developed a virtual peer support group composed of university students from Taiwan. This peer group served as an effective method for developing social connections during social isolation caused by the SARS outbreak. In addition to these findings, in a study conducted by Xiao et al. (2020) during the COVID-19 crisis, it was found that social support improved healthcare providers’ sleep quality which, in turn, reduced feelings of anxiety and improved feelings of self-efficacy toward their job tasks. This is in line with a larger body of research that has demonstrated that social support is a key driver of wellbeing. While social support was found to longitudinally influence wellbeing, and not the reverse (Cacioppo et al., 2010; Jia et al., 2017), other findings have suggested reciprocal relationships between these constructs (Kinnunen et al., 2008; Robitaille et al., 2012). Although these findings provide insight about how social support could serve as a protective factor against the negative impacts of an outbreak or pandemic situation, research is still scarce on how social support influences the mental health and wellbeing of workers in the general population in the current COVID-19 context.

#### Social Participation

Social participation has also been demonstrated by previous research to have a protective effect on mental health and wellbeing. For example, in a study conducted in Australia, it was found that wellbeing and civic participation had a bidirectional longitudinal relationship, in which participants who reported high wellbeing the previous year also demonstrated high civic participation during the next year, and vice versa (Ding et al., 2015). Similarly, Kaplan et al. (2012) found that social participation activities (e.g., civic engagement, volunteering, group membership) were associated with recovery from mental health issues, greater quality of life, and greater meaning in life. Although these findings are insightful about the impact of social participation on wellbeing, there is limited research on the role of social participation as a protective factor during a global pandemic in which individuals must adhere to social distancing measures. Specifically, social participation may have less of a role than other protective factors as participation within one’s community is currently restricted due to social distancing measures. Thus, it is necessary to explore the impact of such participation on individuals’ mental health and wellbeing during the COVID-19 crisis.

#### Trust in Healthcare Institutions

A final factor that has been found to influence mental health and wellbeing is trust in healthcare institutions. Previous research indicates that mistrust in healthcare institutions is associated with increased feelings of psychological distress (Ahnquist et al., 2010) and decreased self-reported health ratings (Armstrong et al., 2006; Mohseni and Lindstrom, 2007; Tokuda et al., 2009). In particular, Tokuda et al. (2009) found that participants across 29 Asian countries were more likely to report good health if they had also reported high levels of trust in the healthcare system. Similarly, Mohseni and Lindstrom (2007) found that low institutional trust in the healthcare system was associated with poor self-reported health and low care-seeking behavior. Thus, while mistrust in the healthcare system may pose a risk to an individual’s mental health and wellbeing, trust in healthcare institutions may serve as a protective factor for these outcomes.

In a study conducted by Shaya et al. (2019), it was found that individuals in Lebanon who trusted their physicians were more likely to comply with the medical advice provided to them. These findings are particularly relevant to the COVID-19 crisis, during which compliance with the preventative policies that have been implemented is especially important. Although Sibley et al. (2020) found an increase in trust for the New Zealand law enforcement and government during the first three weeks of the COVID-19 crisis, there is limited research on the impact of trust in healthcare institutions during a global pandemic. Thus, it is important to examine if individuals trust healthcare institutions within Canada and how this trust (or lack thereof) impacts their mental health and wellbeing.

### Summary of Previous Research

In summary, there are many risk factors and protective factors that could contribute to an individual’s mental health and wellbeing both overall and during an outbreak situation. Although previous research studies provide important insights about these factors, there is limited research on the impact such factors, when considered altogether, may have during a global pandemic, during which strict and long-lasting protective measures have been implemented. As such, the current COVID-19 pandemic may have a unique impact on the risk and resilience factors that either hinder or promote people’s mental health and wellbeing during times of adversity and stress.

## Objectives

The present study aims to examine:

1. If and how multiple potential risk factors, including job insecurity, negative changes to one’s income during the COVID-19 crisis, feelings of vulnerability to contracting the virus, lack of confidence in one’s ability to avoid contracting the virus and compliance with preventative policies (i.e., social distancing measures), are associated with mental health and wellbeing outcomes.
2. The associations of potential resilience factors at multiple ecological levels (i.e., trait resilience, family functioning, social support from friends, social participation, and trust in healthcare institutions) with mental health and wellbeing outcomes.
3. Whether the above-mentioned potential resilience factors act as buffers (i.e., moderators) against the negative impacts of the identified risk factors.

Note that we are using the expression “associated with” and “associations” here given that the presented results are cross-sectional. However, from a conceptual standpoint and based on some studies mentioned above suggesting causality, potential risk and resilience factors will be modeled as impacting wellbeing.

## Materials and Methods

### Participants

Participants were recruited in March 2020 over a period of about one week. Recruitment started approximately one week after the first COVID-19 social distancing measure was recommended by public health authorities in the country. People eligible to complete the online survey were those who: (1) were 18 year of age or older, (2) resided in Canada, and (3) had worked at least 20 h per week (in any job and organization) before the beginning of the COVID-19 crisis. The Qualtrics survey, provided in English, included three attention check questions to ensure participants who were not paying attention (including those who may have been fraudulent participants) were excluded. The final sample included 1,122 participants. Table 1 shows a description of their sociodemographic and work-related backgrounds. As shown in that table, the average age of workers who took part in the study was 39.43 (*SD* = 12.13) with minimum and maximum ages of 18 and 71, respectively. Workers were most likely to be a woman (74.2%), born in Canada (85.5%), cisgender (97.8%), heterosexual (76.3%), able-bodied (81.2%), Caucasian (89.0%), and living in the Province of Ontario (47.8%). Workers were also most likely to have completed an undergraduate degree or a college or trade school diploma/certificate (respectively, 33.7 and 21.0%). In addition, participants were more likely to not have been laid off (80.3%) nor to have experienced a decline in their income and benefits due to COVID-19 (62.3%), and they were likely to have enough money (but no extra savings) before the pandemic occurred (37.5%). Finally, participants had an average of 2.50 people in their household (*SD* = 1.23) and were more likely to not have school-aged children (77.8%).

**TABLE 1:** Characteristics of participants.

Variables	Frequency (n)	Percentage (%)	% missing
**Age in years**			
39.43 ± 12.13 (*M* ± *SD*)			
**Born in Canada**			
Yes	959	85.5	
No	163	14.5	0.0
**Educational level**			
Did not graduate high school	17	1.5	
High school graduate	101	9.0	
Some college or trade school	79	7.0	
College or trade school graduate	236	21.0	
Some university	106	9.4	
University (Bachelor’s degree)	378	33.7	
University (Graduate or professional degree)	204	18.2	0.1
**Lost job temporarily or permanently due to COVID-19**			
Laid off	221	19.7	
Not laid off	901	80.3	0.0
**Experiencing income and benefit changes due to COVID-19**			
Yes	423	37.7	
No	699	62.3	0.0
**Gender**			
Women	832	74.2	
Men	254	22.6	
Non-binary	28	2.5	0.7
**Transgender**			
Yes	13	1.2	
No	1,097	97.8	1.1
**Sexuality**			
Heterosexual	856	76.3	
Minority (e.g., gay, lesbian, bisexual)	245	21.8	1.9
**Having a disability**			
Yes	195	17.4	
No	911	81.2	1.4
**Racialized**			
Yes	104	9.3	
No	999	89.0	1.7
**Having kids that require childcare or are going to school**			
Yes	249	22.2	
No	873	77.8	0.0
**Household income situation before COVID-19**			
Comfortable with extra	395	35.2	
Enough but no extra	421	37.5	
Have to cut back	142	12.7	
Cannot make ends meet	43	3.8	10.8
**Number of people in household**			
2.50 ± 1.23 (*M* ± *SD*)			
**Residing province/territory**			
Alberta	123	11.0	
British Columbia	151	13.5	
Manitoba	49	4.4	
New Brunswick	42	3.7	
Newfoundland and Labrador	21	1.9	
Northwest Territories	2	0.2	
Nova Scotia	87	7.8	
Nunavut	0	0.0	0.0
Ontario	536	47.8	
Prince Edward Island	3	0.3	
Quebec	60	5.3	
Saskatchewan	44	3.9	
Yukon	3	0.3	0.0

### Procedure

The study was approved by Wilfrid Laurier University’s Research Ethics Board (REB #6497). Participants were recruited through social media advertising and a voluntary online panel of workers used by Qualtrics. Social media advertisement consisted of a post that was advertised to Facebook users indicating the inclusion criteria of the study. Researchers also shared the advertisement on their personal Facebook page and community forums where users could share the post within their online circles. The online panel was recruited directly through an invitation on their panel survey platform. Workers interested in participating were directed to the online survey platform where they first filled out a consent form. Those who did not meet the inclusion criteria, or did not consent to taking part in the survey, were thanked for their interest and redirected out of the survey. Those who satisfied all conditions were then redirected to the online survey. Upon completion of the survey, workers who were recruited via social media were given the option to enter a raffle for a $50 dollar gift card. Alternatively, those recruited using Qualtrics’ panel of participants were compensated through their panel company. Finally, a resource list was provided to all participants at the end of the survey which included a list of mental health resources. The survey included a broad range of wellbeing, mental health as well as family and workplace-related questions in order to obtain a holistic portrait of participants’ situation; however, only the data obtained with the measures described in the following section are used in the present article. The median time it took for survey participants to complete the entire survey was 39 min. Although the study includes three measurement waves, the current article presents only the findings from the first wave, which provides early insight into how several resilience factors may buffer the effects of potential risk factors against mental health issues and wellbeing.

### Measures

#### Wellbeing and Mental Health

Three indicators were used to assess workers’ mental health and wellbeing, including measures related to perceived stress, presence and development of distress symptoms (i.e., depression and anxiety), and meaning in life.

##### Stress

To measure perceived stress, an adapted form of the four-item version of Cohen et al. (1994) Perceived Stress Scale (PSS) was adopted. The timeframe used within the scale was altered to measure the stress experienced by workers’ within the first week of the COVID-19 pandemic (e.g., “In the LAST WEEK, how often have you felt that you were unable to control the important things in your life?” and “In the LAST WEEK, how often have you felt that things were going your way?”) The four items were recorded using a five-item scale ranging from 1 (*never*) to 5 (*very often*). Items were coded in such a way that a high score indicated experiences of more stress. The Cronbach’s alpha of the adapted four-item PSS scale indicated an adequate internal consistency within the present study (α = 0.80).

##### Distress

Distress was assessed through a self-reported measure of anxiety and depression symptoms, using Kroenke et al. (2009) Patient Health Questionnaire for Depression and Anxiety (PHQ-4). The PHQ-4 is a brief, but effective, tool used to screen for symptoms related to anxiety and depression. The scale included four items framed within the last week (e.g., “Feeling nervous, anxious or on edge,” “Feeling down, depressed or hopeless”), which were scored on a four-item scale ranging from 1 (*none of the time*) to 4 (*all of the time*). Within the current study, the PHQ-4’s internal consistency was excellent (α = 0.90).

##### Meaning in Life

Questions from the Meaning in Life Questionnaire were adapted to fit the current COVID-19 context (Steger et al., 2006; see also Steger et al., 2008). Four items (e.g., “Like my life is meaningful,” “Like my life has clear purpose”) were contextualized within the current moment so workers would consider their experiences in the present moment when responding. The four items were recorded using a seven-point scale ranging from 1 (*strongly disagree*) to 7 (*strongly agree*). The adapted scale had an excellent Cronbach’s alpha (α = 0.92).

#### Potential Risk Factors

Risk factors that may be associated with lower mental health and wellbeing during the current pandemic were also included. The following scales were used to examine the changes in income workers may have experienced due to COVID-19, their perceived vulnerability to contracting the virus, their lack of confidence in being able to act to prevent contracting the virus, and whether they are complying with preventative measures (i.e., social distancing measures).

##### Income Reduction

Changes in workers’ financial income as a result of the COVID-19 pandemic were measured using a slide scale. Workers were asked the following question, “On the slide scale below, select what your current income/benefits of represent in percent compared to your income/benefits before the coronavirus (COVID-19) crisis affected your employment?” Workers then selected a position on the scale which best represents their change of income relative to that prior to the crisis. On the scale, zero indicated a complete loss of income, 50 indicated the workers’ current income/benefit were equal to 50% of their previous income/benefits, and 100 indicated that the workers’ current income/benefits had not changed (i.e., stayed the same). Before analysis, this variable was reverse coded so higher scores indicated a greater reduction in one’s income due to the crisis.

##### Job Insecurity

To assess job insecurity, four items (i.e., “If my organization suffered a serious crisis, I might lose my job,” “If my organization suffered a serious crisis, I would still get paid until we could reopen,” “If my organization suffered a serious crisis, I would still have my job,” “If my organization suffered a crisis, I would still be covered by my organization’s employee benefits”) from Fowler et al. (2007) Crisis and/or Disaster Preparedness Scale were used. The questions were answered on a four-point scale ranging from 1 (*strongly disagree*) to 4 (*strongly agree*). The positive items were re-coded so higher scores indicated more job insecurity. The four items had a very good internal consistency (α = 0.87).

##### Feeling of Vulnerability to Contracting COVID-19

Workers’ feelings of vulnerability to COVID-19 in relation to both themselves and others (i.e., family, neighbors, friends) was measured using three items from the perceived susceptibility scale proposed by Yoo et al. (2016) in the context of the Middle East respiratory syndrome (MERS) in South Korea. The three items [e.g., “Coronavirus (COVID-19) infection could happen to me,” “Coronavirus (COVID-19) infection could happen to my family”] were adapted to fit the current context of the coronavirus crisis. The items were answered on a five-point scale ranging from 1 (*strongly disagree*) to 5 (*strongly agree*). The internal consistency of the measure was excellent (α = 0.91).

##### Compliance With Social Distancing Measures

As one of the most effective ways of preventing the spread of infectious diseases, complying with recent social distancing policies is highlighted by public health policymakers as crucial to flatten the curve of COVID-19 cases (Centers for Disease Control and Prevention, 2020). In the study, participants were asked “Have you isolated yourself from others (i.e., social distancing) to prevent contaminating others or being contaminated with the coronavirus (COVID-19)?” which was answered using either *no* (1) or *yes* (2).

##### Lack of Confidence in One’s Abilities to Avoid COVID-19

The perception that contracting COVID-19 was unavoidable was explored using three items. The first two were adapted from Rolison and Hanoch’s (2015) and Veldhuijzen et al. (2005) surveys, respectively, on SARS and Ebola (“In general, do you think that people can take actions to prevent getting the coronavirus (COVID-19)?” “How confident are you that you can prevent getting the coronavirus (COVID-19)?”). Each item was answered on a scale from 1 (*not at all confident*) to 5 (*extremely confident*). The third item was adapted from Yoo et al. (2016) survey in the MERS context (“I can figure out how to avoid the coronavirus (COVID-19) infection”), and was answered on a scale from 1 (*strongly disagree*) to 5 (*strongly agree*). A principal component analysis was conducted including these three items suggesting the presence of only one factor (eigenvalue = 1.72). The measure showed satisfying internal consistency (α = 0.63). Although it is lower than other scales, given that Cronbach’s alpha is sensitive to the number of items, it is to be expected that the current scale would obtain a lower value (Black, 1999; Kaplan and Saccuzzo, 2001). In such a context, the mean inter-item correlation is considered to offer a good reliability indicator. As it was 0.36 in the current study, it is within the satisfying range (i.e., 0.20–0.40, Briggs and Cheek, 1986).

#### Potential Resilience Factors

Plausible resilience factors explored in the presented study include trait resilience, family functioning, social support from friends, social participation, and trust in the healthcare institutions.

##### Trait Resilience

Trait resilience was measured using three items (i.e., “I tend to bounce back quickly after hard times,” “I have a hard time making it through stressful events,” “It is hard for me to snap back when something bad happens”) from Smith et al. (2008) Brief Resilience Scale (BRS). Each item was answered on a four-point scale ranging from 1 (*strongly disagree*) to 5 (*strongly agree*). These items were selected given they had the highest factor loadings on average across the multiple validation samples presented in Smith et al. (2008). Within the current study, the three-item measure had a satisfactory Cronbach’s alpha (α = 0.84).

##### Family Functioning

Smilkstein et al. (1982) Family APGAR scale was adopted. The scale consists of five items that measure five parameters of family functioning, including: adaptation, partnership, growth, affection, and resolve. Items (e.g., “I am satisfied that I can turn to my family for help when something is troubling me,” “I am satisfied with the way my family talks over things with me and shares problems with me”) were answered on a seven-point scale ranging from 1 (*almost always*) to 3 (*hardly ever*). The items were reverse-coded to indicate positive family functioning. The Family APGAR scale had a Cronbach’s alpha of 0.90 in the present study, thus showing excellent internal consistency.

##### Social Support

Four items related to the perceived social support from friends found within the Multidimensional Scale of Perceived Social Support scale (MSPSS, Friedlander et al., 2007) were used (e.g., “My friends really try to help me,” “I can count on my friends when things go wrong”). These items were scored on a four-point Likert scale ranging from 1 (*very strongly disagree*) to 7 (*very strongly agree*). These four items had a very high internal consistency (α = 0.94) in the study.

##### Social Participation

To explore workers’ engagement in social activities occurring outside the household a single item measuring the degree of social participation was included. An item from Montpetit et al. (2011) survey was adapted. Workers in the current study were asked “During the LAST WEEK, how often did you participate in social activities outside your home?” which was answered on a four-point scale ranging from 1 (*never*) to 4 (*every day or almost every day*).

##### Trust in Healthcare Institutions

The trust in health care institutions subscale of the Multidimensional Trust in Health Care Systems Scale developed by Egede and Ellis (2008) was included to measure Canadian workers’ trust in our healthcare system. The subscale utilizes three items (i.e., “Health care institutions only care about keeping medical costs down, and not what is needed for my health,” “Healthcare institutions provide the highest quality in medical care,” “When treating my medical problems, health care institutions put my medical needs above all other considerations, including costs”), which were measured on a five-point scale ranging from 1 (*strongly disagree*) to 5 (*strongly agree*). Negative items were re-coded so that high scores indicate higher trust. The subscale had a Cronbach’s alpha of 0.74, thus showing an adequate internal consistency.

#### Control Variables

Demographic questions were included to control for the fact that individual characteristics related to one’s identity and work may influence wellbeing and mental health during the COVID-19 crisis. Control variables included: women vs. men, age, sexual orientation (heterosexual vs. minority), disability, identifying as transgender, being racialized, having children that require child care and/or school-aged children, being born outside of Canada (i.e., migrant), level of education, number of people living in the household, and perception of financial hardship prior to the COVID-19 crisis.

### Data Analysis

Descriptive analysis and univariate correlations were conducted in the SPSS software (v.27, IBM Corp., 1989–2020). The main analyses were conducted in the Mplus software (v.7.31, Muthén and Muthén, 1998–2012) using a structural equation modeling approach in which latent constructs are represented with multiple observed indicators (Wang and Wang, 2019). In all these analyses, whenever a factor or construct was measured by more than one observed variable, it was included in the model as a latent construct, on which all of the observed variables (i.e., measured items) of that factor were loading. The only exception to this was related to the distress construct measured with the PHQ-4, which, in line with previous research (Kroenke et al., 2009; Löwe et al., 2010), was represented by a second-order construct on which two first-order constructs (anxiety and depression) are loading, each represented by two observed variables. In the case of factors measured with a single item, the factor was included in the model directly as an observed variable. After testing each model, several indices of fit providing by the software were examined to assess the adequacy of the tested model: Comparative Fit Index (CFI) and Tucker Lewis Index (TLI) ≥ 0.90; Root Mean Square Error of Approximation (RMSEA) ≤ 0,07; Standardized Root Mean Square Residual (SRMR) ≤ 0.08 (Hooper et al., 2008). Modification indices were requested from the software, providing suggestions that could improve the fit of the model. Modification indices were considered with caution in order to avoid overfitting the model by adjusting it on the basis of these empirically derived modification indices without considering the substantive meaning of model modifications (Schumacker and Lomax, 2010). The Robust Maximum Likelihood (MLR) estimators with robust standard error was used, which is known to be robust to potential data non-normality (Wang and Wang, 2019). In conjunction with MLR, The Mplus software implements the Full-Information Maximum Likelihood (FIML) approach, which is recognized to be one of the best means of dealing with missing values (Enders, 2010). Following recommended practices, whenever the software allowed it, in the current study, auxiliary variables (listed in the Results section) were included in the model using the Mplus “auxiliary (m)” command in order to further reduce potential biases associated with missing values (Enders, 2010).

To address the first research objective, a model was tested in which pathways were included from each of the five risk factors to each of the three wellbeing outcome constructs. To address the second research objective, the risk factors were kept in the models, but this time, we added pathways from each of the five potential resilience factors to each of the wellbeing outcome constructs. This allowed us to test the main effect of resilience factors on wellbeing outcomes. In order to control for demographic variables’ impacts, these tested models included pathways from each of the control variables described above to each of the wellbeing outcome constructs. The model also included correlations between exogenous variables, which is a default in Mplus (Muthen, 2005). These correlations will not be represented in the final model figures in order to simplify graphical representation of the findings.

To address the third research objective, each of the risk factors found to be associated with lower wellbeing constructs was then considered in interaction with each potential resilience factor. While it used to be challenging to test such interactions, the Mplus software now allows the inclusion of interaction terms involving one or two latent factors using the integration algorithm with the Montecarlo integration option (Wang and Wang, 2019). Given the computational requirements of such an algorithm, it proved impossible to test the full model including all potential interactions, all wellbeing outcome constructs and all control variables at once. Thus, a series of smaller models was tested. In these models, only the control variables that were significantly related to wellbeing outcomes in the models above were retained; any non-significant pathways between a control variable and a wellbeing outcome construct was removed. Each test model included: (1) one risk factor latent construct (or observed variable, in case of a single-item measure), (2) one resilience factor latent construct (or observed variable, in case of a single-item measure), and (3) the interaction term between the risk factor and the resilience factor. The model included pathways from each of these three elements to each of the three wellbeing outcome constructs. Note that the software precluded the inclusion of auxiliary variables when testing interactions involving latent factors. However, in one case, the tested interaction involved two observed variables (income reduction and social participation), which did not necessitate the use of the integration algorithm and thus, allowed for the inclusion of the auxiliary variables. In case of significant interaction effects, simple slopes were added into the models using model constraint commands (Stride et al., 2015), to explore the effect of the risk factor at low (one standard deviation below the mean), moderate (mean) and high levels (one standard deviation above the mean) of the resilience factor.

## Results

Table 2 below shows the descriptive statistics and correlations between the main observed variables used in the models. As indicated in this table, most variables were relatively normal; and, the MLR estimator was selected to reduce issues with the few non-normal variables (Wang and Wang, 2019). In terms of missing values, most variables included less than 5% missing values, except job insecurity and social participation. The job insecurity question may not have been answered by participants who had lost their employment because of the crisis, for example. To account for these missing values and reduce biases as much as possible, the FILM approach was used (Wang and Wang, 2019). Auxiliary variables correlated with job insecurity and/or social participation variables and missingness on these variables were also included in the analysis as recommended by Enders (2010). These auxiliary variables included measures focused on feelings (jumbled, conflicted, chaotic, and uneasy) over the last week (McGregor et al., 2001), items from an additional scale of general job security (Kraimer et al., 2005), a variable indicating that one has lost their job temporarily or permanently due to the COVID-19 crisis, and two items focused on financial and occupational wellbeing (Prilleltensky et al., 2015).

**TABLE 2:** Descriptive statistics and correlations between the main study variables (*N* = 1,122).

Measures	1.	2.	3.	4.	5.	6.	7.	8.	9.	10.	11.	12.	13.	14.	15.	16.	17.	18.	19.	20.
**Stress**																				
1. PSS1	–																			
2. PSS2	0.43	–																		
3. PSS3	0.43	0.53	–																	
4. PSS4	0.61	0.53	0.47	–																
**Distress**																				
5. PHQ1	0.58	0.41	0.41	0.58	–															
6. PHQ2	0.60	0.45	0.43	0.64	0.82	–														
7. PHQ3	0.55	0.50	0.45	0.67	0.68	0.73	–													
8. PHQ4	0.49	0.45	0.44	0.61	0.57	0.64	0.78	–												
**Meaning in life**																				
9. M1	−0.35	−0.41	−0.42	−0.44	−0.33	−0.36	−0.46	−0.48	−											
10. M2	−0.40	−0.42	−0.48	−0.46	−0.38	−0.38	−0.49	−0.49	0.78	−										
11. M3	−0.45	−0.46	−0.48	−0.53	−0.47	−0.46	−0.53	−0.51	0.70	0.79	−									
12. M4	−0.35	−0.41	−0.38	−0.43	−0.33	−0.34	−0.45	−0.45	0.75	0.74	0.71	−								
**Income reduction**																				
13. Single item	0.16	0.12	0.19	0.17	0.09	0.10	0.13	0.15	−0.17	−0.19	−0.20	−0.16	−							
**Job insecurity**																				
14. JI1	0.20	0.18	0.17	0.18	0.15	0.15	0.17	0.17	−0.22	−0.22	−0.22	−0.19	0.32	−						
15. JI2	0.22	0.16	0.22	0.23	0.16	0.18	0.17	0.16	−0.25	−0.28	−0.27	−0.21	0.39	0.48	−					
16. JI3	0.21	0.20	0.24	0.24	0.19	0.20	0.22	0.21	−0.31	−0.33	−0.32	−0.25	0.35	0.70	0.67	−				
17. JI4	0.21	0.20	0.24	0.22	0.19	0.20	0.23	0.21	−0.28	−0.30	−0.31	−0.23	0.36	0.52	0.65	0.72	−			
**Vulnerability to COVID-19**																				
18. V1	0.18	0.07	0.12	0.14	0.21	0.18	0.13	0.08	−0.06ns	−0.09	−0.12	−0.08	−0.09	0.04ns	0.08	0.04ns	0.04ns	−		
19. V2	0.17	0.09	0.13	0.11	0.22	0.18	0.11	0.06	−0.05ns	−0.11	−0.13	−0.06	−0.09	0.05ns	0.06ns	0.07ns	0.04ns	0.81	−	
20. V3	0.18	0.09	0.13	0.12	0.20	0.17	0.10	0.07	−0.07	−0.12	−0.14	−0.07	−0.07	0.08	0.05ns	0.07	0.04ns	0.72	0.80	−
**Compliance with social distancing measures**																				
21. Single item	−0.01ns	−0.11	−0.10	−0.05ns	−0.04ns	−0.04ns	−0.06	−0.06ns	0.06ns	0.03ns	0.02ns	0.06ns	0.07	0.06ns	−0.05ns	0.05ns	−0.03ns	−0.04ns	0.04ns	0.00ns
**Lack of confidence**																				
22. LoC1	0.07	0.10	0.07	0.08	0.01ns	0.04ns	0.08	0.11	−0.17	−0.16	−0.18	−0.18	−0.04ns	0.04ns	−0.02ns	0.02ns	0.03ns	0.00ns	−0.02ns	0.00ns
23. LoC2	0.23	0.20	0.21	0.26	0.26	0.25	0.21	0.21	−0.20	−0.21	−0.24	−0.23	−0.08	−0.07	0.07	0.07	0.09	0.28	0.20	0.17
24. LoC3	0.14	0.14	0.14	0.18	0.16	0.15	0.15	0.11	−0.12	−0.18	−0.20	−0.18	−0.01	0.08	0.09	0.13	0.12	0.17	0.16	0.12
**Trait resilience**																				
25. TR1	−0.27	−0.37	−0.25	−0.35	−0.29	−0.33	−0.36	−0.32	0.38	0.40	0.38	0.39	−0.08	−0.09	−0.14	−0.14	−0.15	−0.08	−0.08	−0.10
26. TR2	−0.37	−0.41	−0.30	−0.46	−0.41	−0.46	−0.44	−0.44	0.33	0.35	0.37	0.33	−0.07	−0.15	−0.16	−0.18	−0.20	−0.11	−0.08	−0.11
27. TR3	−0.33	−0.38	−0.24	−0.40	−0.34	−0.38	−0.40	−0.40	0.35	0.37	0.37	0.35	−0.10	−0.13	−0.16	−0.18	−0.18	−0.09	−0.07	−0.07
**Family functioning**																				
28. FF1	−0.14	−0.18	−0.15	−0.23	−0.11	−0.13	−0.20	−0.22	0.31	0.29	0.27	0.30	−0.09	−0.09	−0.10	−0.11	−0.13	−0.08	−0.06ns	−0.07
29. FF2	−0.17	−0.19	−0.17	−0.25	−0.14	−0.15	−0.19	−0.21	−0.27	0.29	0.28	0.28	−0.09	−0.04	−0.09	−0.09	−0.12	−0.09	−0.09	−0.05
30. FF3	−0.13	−0.18	−0.17	−0.20	−0.08	−0.12	−0.20	−0.23	0.34	0.30	0.27	0.31	−0.08	−0.08	−0.11	−0.14	−0.13	−0.01ns	−0.02ns	0.00ns
31. FF4	−0.15	−0.18	−0.16	−0.21	−0.12	−0.12	−0.19	−0.21	0.29	0.31	0.28	0.29	−0.06	−0.06	−0.11	−0.11	−0.15	−0.04ns	−0.02ns	−0.01ns
32. FF5	−0.14	−0.17	−0.16	−0.21	−0.15	−0.15	−0.21	−0.21	0.27	0.29	0.25	0.27	−0.09	−0.05	−0.08	−0.11	−0.12	−0.02ns	−0.02ns	−0.02ns
**Social support from friends**																				
33. SF1	−0.04ns	−0.13	−0.09	−0.07	−0.02ns	−0.04ns	−0.07	−0.11	0.23	0.22	0.20	0.23	0.01ns	−0.00ns	−0.10	−0.08	−0.08	0.00ns	−0.00ns	0.01ns
34. SF2	−0.04ns	−0.14	−0.14	−0.10	−0.02ns	−0.06	−0.09	−0.16	0.24	0.21	0.21	0.26	0.00ns	−0.03ns	−0.10	−0.09	−0.08	0.02ns	0.00ns	−0.01ns
35. SF3	−0.02ns	−0.11	−0.11	−0.05ns	−0.00ns	−0.03ns	−0.07	−0.13	0.21	0.18	0.18	0.22	0.03ns	−0.03ns	−0.08	−0.07	−0.07	0.02ns	0.01ns	0.01ns
36. SF4	−0.00ns	−0.12	−0.09	−0.07	0.00ns	−0.03ns	−0.07	−0.13	0.22	0.18	0.19	0.25	0.01ns	−0.03ns	−0.10	−0.10	−0.10	−0.01ns	−0.01ns	−0.01ns
**Social participation**																				
37. Single item	−0.00ns	0.03ns	−0.00ns	0.06ns	0.02ns	0.01ns	0.03ns	0.02ns	0.04ns	0.04ns	0.04ns	0.03ns	0.00ns	0.04ns	−0.04	0.01ns	0.02ns	−0.03ns	−0.02ns	−0.05ns
**Trust in healthcare institutions**																				
38. THI1	−0.16	−0.15	−0.11	−0.22	−0.11	−0.16	−0.18	−0.21	0.21	0.17	0.17	0.20	−0.06ns	−0.06ns	−0.12	−0.12	−0.13	0.00ns	0.00ns	0.03ns
39. THI2	−0.20	−0.21	−0.18	−0.20	−0.16	−0.18	−0.19	−0.20	0.22	0.25	0.26	0.25	−0.05ns	−0.04ns	−0.14	−0.14	−0.14	−0.05ns	−0.09	−0.08
40. THI3	−0.18	−0.19	−0.19	−0.18	−0.14	−0.12	−0.15	−0.14	0.24	0.24	0.26	0.26	−0.05ns	−0.07ns	−0.11	−0.15	−0.15	−0.08	−0.08	−0.08

*n*	1122	1122	1121	1122	1121	1121	1121	1121	1122	1122	1122	1122	1068	1001	999	998	997	1121	1120	1121
*M*	3.46	2.65	3.25	3.10	2.86	2.61	2.29	2.19	4.89	4.51	4.30	4.93	16.74	2.80	2.72	2.65	2.58	4.29	4.33	4.40
*SD*	1.22	.94	.94	1.19	1.10	1.14	1.11	1.09	1.60	1.67	1.72	1.68	29.44	0.96	.97	.96	1.01	.76	.72	.66
Minimum	1	1	1	1	1	1	1	1	1	1	1	1	0	1	1	1	1	1	1	1
Maximum	5	5	5	5	4	4	4	4	7	7	7	7	100	4	4	4	4	5	5	5
Skewness	−0.34	0.09	−0.18	−0.05	−0.30	−0.04	0.33	0.46	−0.75	−0.42	−0.29	−0.78	1.70	−0.41	−0.11	−0.06	0.10	−1.29	−1.22	−1.15
Kurtosis	−0.50	−0.15	−0.20	0.81	−1.36	−1.43	−1.23	−1.10	−0.21	−0.74	−0.89	−0.26	1.73	−0.75	−1.07	−0.99	−1.15	2.67	2.69	2.74

**Measures**	**21.**	**22.**	**23.**	**24.**	**25.**	**26.**	**27.**	**28.**	**29.**	**30.**	**31.**	**32.**	**33.**	**34.**	**35.**	**36.**	**37.**	**38.**	**39.**	**40.**

**Stress**																				
1. PSS1																				
2. PSS2																				
3. PSS3																				
4. PSS4																				
**Distress**																				
5. PHQ1																				
6. PHQ2																				
7. PHQ3																				
8. PHQ4																				
**Meaning in life**																				
9. M1																				
10. M2																				
11. M3																				
12. M4																				
**Income reduction**																				
13. Single item																				
**Job insecurity**																				
14. JI1																				
15. JI2																				
16. JI3																				
17. JI4																				
**Vulnerability to COVID-19**																				
18. V1																				
19. V2																				
20. V3																				
**Compliance with social distancing measures**																				
21. Single item	–																			
**Lack in confidence**																				
22. LoC1	0.05ns	–																		
23. LoC2	−0.09	0.41	–																	
24. LoC3	−0.05ns	0.24	0.42	–																
**Trait resilience**																				
25. TR1	0.03ns	−0.06ns	−0.17	−0.15	–															
26. TR2	0.08	−0.07	−0.21	−0.13	0.54	–														
27. TR3	0.03ns	−0.11	−0.18	−0.12	0.67	0.71	–													
**Family functioning**																				
28. FF1	0.00ns	−0.09	−0.10	−0.09	0.19	0.16	0.18	–												
29. FF2	0.02ns	−0.04ns	−0.09	−0.09	0.21	0.18	0.21	0.65	–											
30. FF3	−0.00ns	−0.08	−0.08	−0.06	0.20	0.17	0.22	0.63	0.63	–										
31. FF4	−0.02ns	−0.11	−0.09	−0.09	0.22	0.18	0.20	0.64	0.72	0.65	–									
32. FF5	−0.02ns	−0.06ns	−0.10	−0.10	0.21	0.15	0.18	0.55	0.62	0.61	0.70	–								
**Social support from friends**																				
33. SF1	0.01ns	−0.11	−0.10	−0.04	0.13	0.12	0.16	0.32	0.28	0.29	0.30	0.22	–							
34. SF2	−0.00ns	−0.11	−0.11	−0.05ns	0.14	0.15	0.16	0.25	0.22	0.25	0.24	0.17	0.82	–						
35. SF3	0.03ns	−0.13	−0.10	−0.03ns	0.13	0.15	0.16	0.26	0.22	0.24	0.24	0.17	0.74	0.76	–					
36. SF4	0.01ns	−0.10	−0.08	−0.04ns	0.16	0.14	0.16	0.25	0.10	0.22	0.22	0.16	0.73	0.78	0.86	–				
**Social participation**																				
37. Single item	−0.07	−0.04ns	−0.03ns	−0.00ns	0.01ns	−0.02ns	−0.06ns	−0.05ns	−0.04ns	−0.03ns	−0.03ns	−0.01ns	0.09	0.05ns	0.08	0.08	–			
**Trust in healthcare institutions**																				
38. THI1	0.07	−0.17	−0.14	−0.13	0.12	.18	0.20	0.13	0.09	0.15	0.11	0.09	0.11	0.14	0.14	0.15	−0.07	–		
39. THI2	0.00ns	−0.15	−0.19	−0.15	0.20	0.20	0.19	0.17	0.16	0.18	0.17	0.15	0.11	0.14	0.09	0.11	−0.03ns	0.43	–	
40. THI3	0.03ns	−0.15	−0.20	−0.19	−0.14	0.10	0.11	0.21	0.20	0.19	0.19	0.15	0.15	0.17	0.14	0.15	−0.01ns	0.44	0.60	–

*n*	1,121	1,120	1,121	1,122	1,122	1,122	1,121	1,118	1,118	1,121	1,120	1,119	1,119	1,121	1,121	1,121	970	1,121	1,121	1,122
*M*	1.86	2.31	2.87	2.62	3.50	3.04	3.19	2.54	2.35	2.52	2.35	2.43	5.26	5.21	5.45	5.40	1.31	3.51	3.43	3.14
*SD*	0.35	1.06	1.17	0.97	0.99	1.12	1.11	0.63	0.68	0.63	0.71	0.66	1.31	1.40	1.44	1.41	0.65	1.04	0.95	1.01
Minimum	1	1	1	1	1	1	1	1	1	1	1	1	1	1	1	1	1	1	1	1
Maximum	2	5	5	5	5	5	5	3	3	3	3	3	7	7	7	7	4	5	5	5
Skewness	−2.01	0.65	0.25	0.37	−0.51	−0.01	−0.27	−1.06	−0.57	−0.96	−0.61	−0.74	−0.93	−0.96	−1.30	−1.20	2.09	−0.51	−0.48	−0.27
Kurtosis	2.41	−0.19	−0.80	−0.28	−0.32	−0.99	−0.86	0.03	−0.74	−0.16	−0.84	−0.55	1.00	0.87	1.53	1.41	3.57	−0.30	−0.23	−0.49

*All correlations were significant at p ≤ 0.05 or less, except values with the “ns” indication, which were not significant.*

**FIGURE 2:**
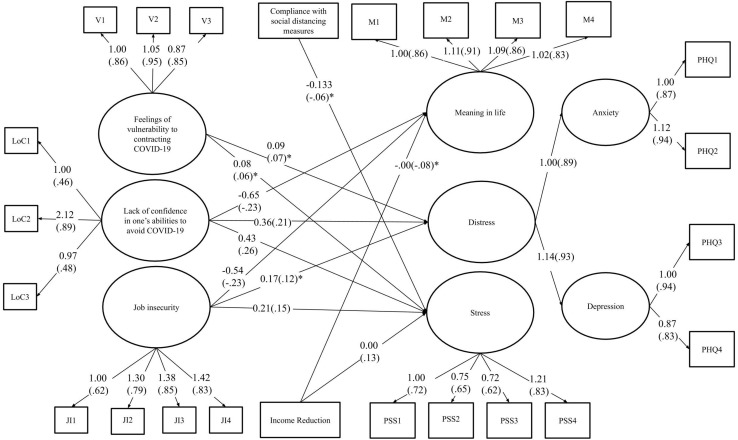
Final model of the pathways between potential risk factors and wellbeing outcomes. The estimates in brackets are standardized while those not presented in brackets are unstandardized. All connections illustrated are significant at *p* ≤ 0.001, except for those with an asterisk (*) which indicates that the effect is significant at *p* ≤ 0.05. Control variables were included when conducting the analysis, but were excluded from the figure for reading ease.

The first structural equation model that was tested included all the risk factors and their pathways to each wellbeing outcome constructs. The model [χ^2^(416) = 1195.76, TLI = 0.95; CFI = 0.92; RMSEA = 0.04 (90% CI [0.04, 0.04]); SRMR = 0.03] showed good fit. Modification indices suggested the addition of a correlational link between two items of the job insecurity construct, which we decided to add, given that the two concerned items were clear opposites of each other and thus, likely highly negatively linked, i.e., “If my organization suffered a serious crisis, I might lose my job,” and “If my organization suffered a serious crisis, I would still have my job”. The model was tested again with this addition. The model fit [χ^2^(415) = 1116.40, TLI = 0.96; CFI = 0.93; RMSEA = 0.04 (90% CI [0.04, 0.04]); SRMR = 0.03] was slightly improved and deemed satisfactory. Figure 2 shows the significant pathways of the final model. As shown, all observed variables loaded as expected on their respective latent construct. The model included the following pathways between risk factors and wellbeing outcome constructs: (1) positive pathways to stress from income reduction, job insecurity, lack of confidence in avoiding COVID-19, and vulnerability to COVID-19; (2) positive pathways to distress from income reduction, job insecurity, lack of confidence in avoiding COVID-19, and vulnerability to COVID-19; and (3) negative pathways to meaning in life from income reduction, job insecurity, and lack of confidence in avoiding COVID-19. The model also included a negative pathway from social distancing to stress, indicating lower levels of stress among participants practicing social distancing.

**FIGURE 3:**
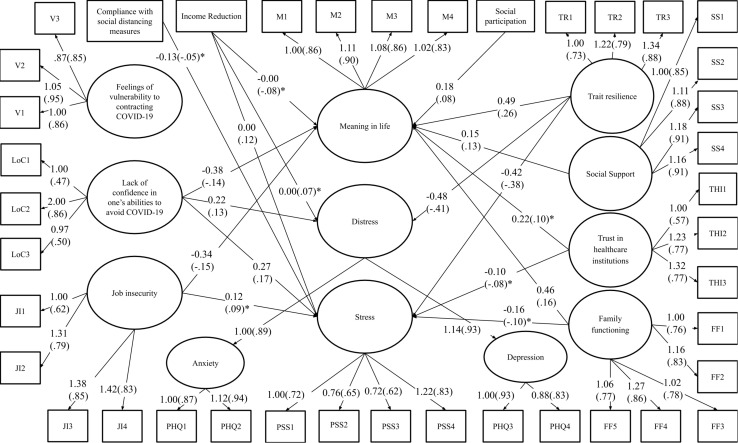
Final model of the pathways between resilience and risk factors (direct effects only) and wellbeing outcomes. The estimates in brackets are standardized while those not presented in brackets are unstandardized. All connections illustrated are significant at p ≤ .001, except for those with an asterisk (*) which indicates that the effect is significant at p ≤ .05. Control variables were included when conducting the analysis, but were excluded from the figure for reading ease.

The final model was used as the basis for the next model to be tested, in which each of the potential resilience factors was added, including a pathway between these factors and each wellbeing outcome construct. The model [χ^2^(986) = 2214.25 TLI = 0.95; CFI = 0.94; RMSEA = 0.03 (90% CI [0.03, 0.04]); SRMR = 0.03] showed excellent fit. The final model is shown in Figure 3. In terms of the significant pathways between risk factors and wellbeing outcome constructs, they were overall the same as in Figure 2, except that vulnerability to COVID-19 was no longer significantly associated with distress and stress, and the pathway between job insecurity and distress was also not significant. With regards to potential resilience factors, the following were significant: (1) negative pathways to stress from trait resilience, family functioning, and trust in healthcare institutions; (2) negative pathways to distress from trait resilience; and (3) positive pathways to meaning in life from trait resilience, family functioning, support from friends, social participation, and trust in healthcare institutions.

A series of 20 models were then run specifically to examine the interaction effect between each of the four risk factors and each of the five resilience factors. Given its association with less stress, social distancing was found not to be a risk factor in the analysis above (see Figure 2), and as such, it was not considered in this interaction analysis. As shown in Table 3, most of the interaction effects were not significant, except for: (1) the interaction effect between income reduction and social support from friends on meaning in life; (2) the interaction effect between job insecurity and trait resilience on meaning in life; (3) the interaction effect between job insecurity and social support from friends on stress; and (4) the interaction effects between lack of confidence in avoiding COVID-19 and trait resilience on stress and meaning. The other interaction effects were not significant, and the pattern of main effects found in Figure 3 was overall confirmed.

**TABLE 3:** Findings from the moderation analysis testing the interaction between each risk and each resilience factor.

	Using trait resilience as the resilience factor			Using family functioning as the resilience factor			Using social support from friends as the resilience factor			Using social participation as the resilience factor			Using trust in healthcare institutions as the resilience factor		
	DV: Stress^a^	DV: Distress^b^	DV: Meaning^c^	DV: Stress^a^	DV: Distress^b^	DV: Meaning^c^	DV: Stress^a^	DV: Distress^b^	DV: Meaning^c^	DV: Stress^a^	DV: Distress^b^	DV: Meaning^c^	DV: Stress^a^	DV: Distress^b^	DV: Meaning^c^
	*B*	*B*	*B*	*B*	*B*	*B*	*B*	*B*	*B*	*B*	*B*	*B*	*B*	*B*	*B*
IV: Risk factor: Income reduction	0.003***	0.001	−0.005***	0.004***	0.002*	−0.006***	0.004***	0.002*	−0.007***	0.004***	0.002**	−0.007***	0.004***	0.002*	−0.006***
MOD: Resilience factor	−0.376***	−0.387***	0.505***	−0.185***	−0.143***	0.429***	−0.113***	−0.087**	0.445***	−0.003	−0.032	0.141*	−0.184***	−0.130***	0.343***
Interaction term	0.000	0.000	−0.002	0.001	0.000	0.000	0.001	0.001	−0.003*	−0.001	0.001	0.002	0.000	0.000	0.001

IV: Risk factor: Job insecurity	0.132***	0.085**	−0.340***	0.170***	0.126***	−0.356***	0.174***	0.129***	−0.354***	0.193***	0.148***	−0.407***	0.166***	0.124***	−0.366***
MOD: Resilience factor	−0.353***	−0.375***	0.494***	−0.157***	−0.121***	0.400***	−0.081***	−0.060*	0.355***	−0.001	−0.013	0.156**	−0.156***	−0.108***	0.311***
Interaction term	0.016	−0.024	0.078*	0.012	−0.002	−0.039	0.058*	0.036	−0.080	−0.043	−0.059	0.086	−0.004	0.020	0.059

IV: Risk factor: Vulnerability to COVID-19	0.064**	0.070**	−0.049	0.082**	0.091***	−0.064	0.088***	0.094***	−0.074*	0.090**	0.082**	−0.063	0.078**	0.087***	−0.056
MOD: Resilience factor	−0.376***	−0.388***	0.543***	−0.177***	−0.134***	0.439***	−0.097***	−0.072**	0.394***	0.006	−0.006	0.161**	−0.180***	−0.122***	0.369***
Interaction term	0.042	0.022	0.014	0.032	−0.014	0.019	−0.010	−0.003	0.010	0.067	0.050	−0.001	0.003	0.009	−0.060

IV: Risk factor: Lack of confidence in avoiding COVID-19	0.158***	0.122***	−0.236***	0.215***	0.186***	−0.306***	0.218***	0.190***	−0.295***	0.244***	0.209***	−0.390***	0.199***	0.178***	−0.279***
MOD: Resilience factor	−0.336***	−0.357***	0.483***	−0.152***	−0.116***	0.405***	−0.071**	−0.051	0.361***	−0.010	−0.021	0.174**	−0.122***	−0.071*	0.285***
Interaction term	0.057*	0.044	−0.105*	0.000	−0.042	0.062	0.015	0.033	−0.051	−0.058	−0.075	0.081	0.006	−0.019	−0.023

All analyses conducted with the full sample (N = 1022), except when involving either the Income reduction or the Social participation variables, for which cases with missing values on these variables had to be excluded, leaving the analytical sample to 1,068 and 970, respectively. V, independent variable; DV, dependent variable; MOD, moderator. ^a^Controlling for age, gender, living with disability, being born outside Canada, financial situation before the COVID-19 crisis, social distancing. ^b^Controlling for age, gender, education level, living with disability, being born outside Canada, financial situation before the COVID-19 crisis. ^c^Controlling for age, gender, number of people in the household, living with disability, financial situation before the COVID-19 crisis. ***p ≤ 0.001, **p ≤ 0.01, *p ≤ 0.05.

The simple slopes of the significant interactions were explored. Several different patterns of simple slopes were identified, as shown in Table 4. First, for the interactions between income reduction and social support from friends on meaning in life, at a lower value of social support the effect of income reduction was not significant, while the effect was negative and significant at higher values of social support from friends. A similar pattern was found for the effect of lack of confidence in avoiding COVID-19 on meaning, which was found to be non-significant at a lower value of trait resilience, but significant and negative at higher values of trait resilience.

**TABLE 4:** Simple slopes of risk factor effects at low, moderate and high values of resilience factors in cases of significant interactions between risk and resilience factors.

	Using trait resilience as the resilience factor			Using social support from friends as the resilience factor		
	DV: Stress^a^	DV: Distress^b^	DV: Meaning^c^	DV: Stress^a^	DV: Distress^b^	DV: Meaning^c^
	*B*	*B*	*B*	*B*	*B*	*B*
**Effect of income reduction on DV at**						
−1 *SD* of the resilience factor	Non-significant interaction	Non-significant interaction	Non-significant interaction	Non-significant interaction	Non-significant interaction	−0.003
*M* of the resilience factor						−0.007***
+1 *SD* of the resilience factor						−0.010***
**Effect of job insecurity on DV at**						
−1 *SD* of the resilience factor	Non-significant interaction	Non-significant interaction	−0.418***	0.115**	Non-significant interaction	Non-significant interaction
*M* of the resilience factor			−0.340***	0.174***		
+1 *SD* of the resilience factor			−0.262***	0.232***		
**Effect of lack of confidence in avoiding COVID-19 on DV at**						
−1 *SD* of the resilience factor	0.101**	Non-significant interaction	−0.132	Non-significant interaction	Non-significant interaction	Non-significant interaction
*M* of the resilience factor	0.158***		−0.236***			
+1 *SD* of the resilience factor	0.215***		−0.341***			

Only the significant interaction effects from Table 3 were further explored in this Table. All analyses conducted with the full sample (N = 1022), except when involving Income reduction, for which cases with missing values on that variables had to be excluded, leaving the analytical sample to 1068. IV, independent variable; DV, dependent variable. ^a^Controlling for age, gender, living with disability, being born outside Canada, financial situation before the COVID-19 crisis, social distancing. ^b^Controlling for age, gender, education level, living with disability, being born outside Canada, financial situation before the COVID-19 crisis. ^c^Controlling for age, gender, number of people in the household, living with disability, financial situation before the COVID-19 crisis. ***p ≤ 0.001, **p ≤ 0.01, *p ≤ 0.05.

Another pattern was found for the effect of job insecurity on stress. This effect was positive (i.e. detrimental) and significant at all values of social support from friends, but increased in magnitude (i.e., stronger association with stress) as values of social support from friends increased. A similar pattern was found for the effect of lack of confidence in avoiding COVID-19 on stress. The effect was found to be positive (i.e., detrimental) at all levels of trait resilience, but it increased in magnitude (i.e., stronger association with stress) as trait resilience increased.

A final pattern—the only one consistent with a buffering effect understanding of resilience factors—was found for the interaction between job insecurity and trait resilience on meaning in life. While the negative effect of job insecurity was found to be significant at all levels of trait resilience, it actually decreased in magnitude (i.e., weaker association with meaning in life) as trait resilience increased.

## Discussion

The purpose of the present study was to explore if, and how, several risk and resilience factors were associated with mental health and wellbeing outcomes among workers in the early stages of the COVID-19 crisis. Overall, it was found that each of the tested potential risk factors, except social distancing, was related to lower mental health and wellbeing, while each of the resilience factors was positively related to more positive mental health and wellbeing. The effects of risk and resilience factors were most often main effects (i.e., not interactional).

### Risk Factors

Overall, the findings corroborate those of previous literature. In particular, the results demonstrating that reduction to one’s income and job insecurity during the COVID-19 crisis were associated with higher levels of stress are in accordance with findings from several comprehensive literature reviews indicating that such factors can enhance psychological distress and mental health issues (Virtanen et al., 2005; Mihashi et al., 2009; Nieuwenhuijsen et al., 2010; Schmidt et al., 2014). According to Statistics Canada (2020), job security and income were indeed threatened by the COVID-19 pandemic, thus, our results—indicating that job insecurity was associated with lower wellbeing—are important to consider.

Higher levels of stress were also associated with stressors more directly related to the virus itself, such as stronger feelings of vulnerability to contracting COVID-19, and lack of confidence in avoiding COVID-19. These findings are similar to those from previous research indicating that fear of oneself or important others in one’s life becoming infected with a disease can induce feelings of anxiety, in addition to other negative mental and physical health outcomes (Maunder et al., 2003; Hawryluck et al., 2004; Reynolds et al., 2008; Brooks et al., 2020).

In addition to these findings, income reduction, job insecurity, and lack of confidence in avoiding COVID-19 were also associated with higher distress and lower meaning in life. To our knowledge, the current study is one of the very few empirical explorations of meaning in life, an important yet often neglected aspect of wellbeing, in the context of a pandemic (Steger et al., 2013). The fact that the findings suggest that even in the very first weeks of the crisis, COVID-19 related stressors are associated with lower momentaneously-perceived meaning in life is concerning given that lower meaning in life has been found to be associated with less capacity to adapt to disaster situations (i.e., tornadoes, Weber et al., 2019). Thus, lower meaning in life in the first few weeks after the COVID-19 crisis started may actually put people on the path toward poorer adaptation in the future, although longitudinal research is needed.

Interestingly, the current model demonstrated a trend of lower stress levels among participants complying with social distancing recommendations. These findings are contrary to previous research which indicates that quarantining and social distancing measures have negative impacts on one’s mental health and well-being (Hawryluck et al., 2004; Reynolds et al., 2008). However, as the study was conducted during the first two weeks after the Canadian government had implemented social distancing measures, it could be that the negative impacts of social distancing measures had not yet been experienced by many individuals. In a study by Hawryluck et al. (2004), it was found that negative mental health outcomes, such as increased symptoms of post-traumatic stress disorder, were seen more frequently in individuals who had been quarantined for greater than 10 days. Thus, individuals from the current study that participated within the first 10 days of implementation of preventative measures, may not have experienced the full impact of social distancing yet.

### Resilience Factors

In regard to resilience factors, all of those considered displayed a main positive effect on one or more of the mental health and wellbeing outcomes. Overall, meaning in life was positively associated with several protective factors, including trait resilience, better family functioning, higher social support from friends, social participation, and trust in healthcare institutions. In addition, lower stress was associated with both better family functioning and trust in healthcare institutions, and lower distress was associated with higher trait resilience. Of the five resilience factors, trait resilience seemed to be one of the most important as higher trait resilience was the only resilience factor found to be associated with each of the three considered wellbeing outcomes constructs. These findings are in line with those from previous research with several populations and in various traumatic situations, including the COVID-19 crisis (Hu et al., 2015; Nam et al., 2016; Kavčič et al., 2020). In particular, Kavčič et al. (2020) found trait resilience to be a protective factor against mental health issues and perceived stress for Slovene adults during the COVID-19 crisis. Extending Kavčič et al. (2020) findings, the current results demonstrate that trait resilience is associated with greater feelings of meaning in life, and lower distress.

These findings are also supported by those focused on an intervention aimed at promoting feelings of resilience and meaning among individuals living with chronic illnesses during the SARS outbreak. More specifically, an intervention implemented one month after the SARS outbreak utilized the Strength-Focused and Meaning-Oriented Approach for Resilience and Transformation (SMART) model and found that upon teaching participants how to enhance feelings of resilience and meaning, these individuals reported a decrease in mental health issues and symptoms, as well as more positive cognitive appraisals of their social and personal experiences during the SARS outbreak up to one month later (Ng et al., 2006). These findings suggest that resilience can be taught in the context of pandemics, and as such, lower resilience and its associations with poorer wellbeing, as found in the current study, are not inevitable.

Although trait resilience was an important factor in reducing stress and distress and increasing feelings of meaning in life, the results from the current study support the use of an ecological approach when understanding the impact of resilience factors on an individual’s mental health and wellbeing. In line with research by Ungar and Theron (2020), the present findings demonstrate that resilience is an integrative process made up of psychological, social, and systemic factors. For example, results from the current study suggest that family functioning could be a protective factor against mental health issues and poor wellbeing as it was associated with lower stress and greater feelings of meaning in life. This aligns with previous research conducted during disaster situations (e.g., hurricane, tsunami), according to which positive family relationships serve to reduce mental health symptoms (e.g., depression, PTSD) among children (Wickrama and Kaspar, 2007; Kronenberg et al., 2010). Further, the presence of a positive relationship with even one family member has been shown to be enough to buffer the negative impacts of psychological distress caused by unfavorable circumstances (Vakrat et al., 2018; Davies et al., 2019). In the context of COVID-19, family functioning is especially important to consider as many families are spending a greater amount of time together due to preventative policies, such as social distancing.

Although the associations of social support from friends and social participation with stress and distress were not significant in the current study, previous research does support such associations. For example, in a study by Glass et al. (2009), it was found that social support from friends was associated with low psychological distress among Hurricane Katrina survivors. In addition to these findings, a study by Sanders et al. (2004) supports the association between social support from friends and feelings of meaning in life as seen in the present findings. Specifically, Sanders et al. (2004) found that older adults who were forced to relocate due to a hurricane reported increased mental health symptoms (i.e., depression) and reduced feelings of meaning in life as a result of not having friends in their new community. Although the research studies discussed above were conducted during disaster situations (e.g., hurricane), the unique circumstances instilled by the COVID-19 crisis (i.e., social distancing measures) may limit an individuals’ ability to utilize social resources, such as support from friends and social participation, as protective factors for their mental health and wellbeing. As such, this could explain why the relationship between these resilience factors and the wellbeing outcomes related to lower stress and distress were not significant in the present study.

Resilience processes operate on the basis of connections between several levels of the social ecology, and several researchers have recognized this very clearly. For example, Ager (2013) cited Eggerman and Panter-Brick (2010) who wrote “there is no health without mental health, no mental health without family unity, no family unity without work, dignity, and a functioning economy, and no functioning economy without good governance” (p. 83). Our results, showing correlations between the multiple resilience factors, are entirely aligned with such a perspective. Of particular importance at the highest level of the social ecology in our study are health care institutions. As such institutions have experienced immense pressure due to the COVID-19 crisis, it is necessary to understand the role of trust as a resilience factor, which could be promoted or eroded by the extensive and persistent challenges posed by a global pandemic. In a national Canadian survey, it was found that trust in one’s local health authority or medical health officer increased from 79% the week before preventative policies were implemented to 87% the week after, indicating a high level of trust in healthcare institutions and personnel among Canadians in a pandemic (Elflein, 2020). Based on our findings, such high trust is likely beneficial for Canadian workers.

### Unexpected (Reversed) Buffering Effects

In studies on trait resilience and similar concepts (e.g., hardiness) as well as social support (Cohen and Wills, 1985; Beasley et al., 2003; Schiff et al., 2010; Kaniasty et al., 2020) an important question that researchers have been asking themselves is: are these factors having direct effects on wellbeing, independently of the levels of exposition to risks and adverse situations, or do they have interactive, buffering effects specifically associated with reduced impacts of stressors and adverse situations on wellbeing and mental health? As indicated above, all of the five considered potential resilience factors emerged as having positive main relationships on at least one of the wellbeing outcomes constructs, providing support to a direct effect model of protective influence. This is consistent with what others have called compensatory effects of resilience factors (Zimmerman et al., 2013), in which increased levels of resilience factors, although not reducing per se the negative impacts of stressors and risks, have beneficial impacts on wellbeing, thus, in some way compensating for the detrimental effects of stressors and risks. Our findings, mostly highlighting direct effects rather than buffering effects, are also aligned with research on the role of social support in the context of disaster situations, in which many studies have found direct effects while few studies have identified buffering effects (as reviewed by Kaniasty et al., 2020). In the COVID-19 context, from a practice-based perspective, the direct effects of resilience factors suggest that interventions targeting the development of trait resilience, family functioning, social support from friends, social participation, and trust in health institutions may lead to positive impacts that could help counterbalance the negative impacts of the identified risk factors on wellbeing, but that overall, would not directly prevent these negative impacts from happening.

Only one of the identified significant interaction effects was aligned with a buffering effect conceptualization (i.e., the interaction effect of job insecurity and trait resilience), in which higher trait resilience seemed to protect against—or reduce—the negative impact of job insecurity on meaning in life. This is consistent with several previous research studies suggesting that people who initially have higher inner resilience skills are better equipped to deal with major stressors, from war-related trauma exposure (Fino et al., 2020) to natural disasters (Quan et al., 2017) and pandemics (Kavčič et al., 2020). As such, these individuals would be less impacted by these stressors.

Interestingly, in addition to main effects, a few of the factors expected to positively buffer the effects of the identified risk factors were found to interact with stressors in a negative way, nuancing preconceived ideas of what is considered positive and negative in times of crisis. Trait resilience and social support from friends interacted with some of the identified risk factors (i.e., income reduction, job insecurity, lack of confidence in avoiding COVID-19) in a negative way. Specifically, the pattern of results suggests that high levels of these potential resilience factors could indeed be maladaptive for some aspects of wellbeing, amplifying the detrimental effects of COVID-19 related risk factors. In contrast, for people with lower levels of these resilience factors, the associations between these risk factors and wellbeing were weaker. In the past, a few researchers have identified this “reversed buffer effects” pattern (e.g., Antonucci et al., 2010; Kaniasty et al., 2020). When it comes to social support, it is possible that the received support was not appropriate (Antonucci et al., 2010) or relevant given the uncertain and completely unprecedented nature of the crisis. Furthermore, as stated by Kaniasty et al. (2020, p. 345), “having many social linkages within a community severely affected by a disaster could also be a liability, not just an asset”. It is possible that stronger social support indicates a larger number of people in one’s network to be worried about with regards to their safety and adjustment in the COVID-19 context. Indeed a study conducted after Hurricane Katrina suggests that people who were more socially embedded in their community before the event experienced a certain level of burden associated with the expectation that they would offer support, and it was found to contribute to stress (Weil et al., 2012).

When it comes to resilience, Williams et al. (2017) have recently highlighted some potential negative side effects too. For example, when discussing resilience to adversity in an organizational context, these authors mentioned that “resilience assists actors in persisting in activities despite hardship” (Williams et al., 2017, p. 757). It may be the case that in the context of the COVID-19 crisis, that is a resolutely new and unprecedented context, it would better serve people to adjust their expectations and behaviours, rather than to persist with their usual lifestyles and routines. Further research, also in an organizational context, found that the impact of workplace bullying on employee wellbeing was moderated by trait resilience in such a way that more resilient people experienced more negative effects (Annor and Amponsah-Tawiah, 2020). These authors referred to the fact that trait resilience is often associated with reliance on active coping, which may not be useful in situations where people have limited control, and it is plausible to assume the COVID-19 is such a situation. In such contexts, an over-reliance on one’s inner capacities and strengths associated with active coping could lead to the depletion of one’s internal resources (Annor and Amponsah-Tawiah, 2020). From that perspective, it is critical for decision-makers at all levels of society not only to value inner resilience, but also to support individuals’ ability to utilize environmental resilience resources during a pandemic situation.

Another potential interpretation of the reverse buffering effects that we found is that the novelty of the COVID-19 crisis, given that it began only a few weeks before the survey, may have influenced these results.

### Limitations

The limitations associated with the study relate mostly to the cross-sectional nature of the present findings, and the time at which the data was collected. The study provided a nuanced snapshot into the lives of Canadian workers one to two weeks after social distancing measures were implemented. Given that the data was collected very rapidly after the COVID-19 crisis started, our findings may reflect participants’ early levels of adjustment rather than long-term trends. It is possible that more time for participants to adjust to their “new normal” is needed before buffering mechanisms (i.e., significant moderation effects of resilience factors) on wellbeing can actually unfold and be observed. While the study included additional survey waves two weeks and two months later, the analysis for this article only focuses on the first wave, and as such, directionality and causality of the effects cannot be ascertained. Of the three requirements that need to be established to determine causality (Chambliss and Schutt, 2013), the presented paper establishes a correlation between wellbeing outcomes and risk and resilience factors variables. However, temporal precedence was not examined in this study as multiple time points would need to be analyzed. While our hypothesized models operate under the assumption that constructs thought to be resilience and risk factors are impacting wellbeing, and not the reverse, wellbeing could impact the levels of some of the self-reported risk or protective factors. For example, levels of wellbeing (i.e., depressive symptoms) at one time point has been found to predict later perceived level of family functioning (Lorenzo-Blanco et al., 2017). Future publications will allow to examine such longitudinal relationships between constructs through incorporating data from each of the study’s three waves. The third condition for causality is non-spuriousness, and it also cannot be completely ensured in the current article. It is possible, for example, that one’s level of optimism could simultaneously positively influence their levels of wellbeing (e.g., Mäkikangas et al., 2004) and their feelings of vulnerability to COVID-19 (e.g., Park et al., 2020). Longitudinal designs including additional variables as controls could help examine the roles of such potential third variables.

In accordance with the fact that women are more likely in general to participate in surveys (Moore and Tarnai, 2002), 74% of the respondents in our sample were women. This may have impacted the results since women are more likely to experience job insecurity as shown in the literature reviewed by Landsbergis et al. (2014). Further, workers that are women may have additional demanding roles as mothers (Wang and Patten, 2001). This might, in turn, lead to additional stressors that may have interacting effects with COVID-19 crisis risk factors on wellbeing.

The design of the study prevents us from generalizing the results to all Canadian workers. For example, as the study was open to all Canadian adults who were working before the COVID-19 crisis began, the analysis does not specifically explore the in-depth experiences of employees in precarious employment conditions or those who were not working before the crisis started. These segments of the populations, whose wellbeing may already be fragilized in less uncertain times, may have been more affected by the crisis than the current study’s workers (Kantamneni, 2020). Furthermore, the survey was only available online, which may have prevented individuals with limited internet access from participating, especially those in precarious situations, and those individuals from particularly marginalized communities (Vosko, 2013; Robinson et al., 2015; Canadian Radio-television and Telecommunications Commission, 2018, 2019).

In addition, as short measurement scales were used to prevent response fatigue, future research with longer measurement scales may be useful to validate the findings obtained in the current study. In particular, future research should focus on the counterintuitive results related to the relative absence of positive buffering effects among resilience factors. Finally, the higher, although still relatively limited, levels of missing values for measures related to job insecurity and social participation may have impacted the results.

### Implications

The findings of the present study advance the current knowledge about the likely impacts of the COVID-19 crisis, and provide insights about the risk and resilience factors that influence individuals’ mental health and wellbeing. In particular, the findings could be used to inform recommendations for service providers and policymakers about the factors to target with interventions for mental health and wellbeing. In addition to the financial aid currently being provided by the Canadian government, there are other areas for improvement in terms of workers’ job security, social support, feelings of self-efficacy in controlling the virus, family functioning, and social participation.

First, social media may be capitalized on to help workers cope with job insecurity and provide them with social support. As stated by the World Health Organization (2017), social media “may be used to engage the public, facilitate peer-to-peer communication, create situational awareness, monitor and respond to rumors, public reactions, and concerns during an emergency, and facilitate local-level responses.” Aside from the benefits social media provides officials in disseminating information about the pandemic and preventative measures (O’Brien et al., 2020), peer-to-peer communication on social media provides a particular aid for those living through these unprecedented times. For instance, workers would have a place to voice their experiences and share resources. Employers may also use social media and other technological tools to communicate transparently to their employees about the effects of the crisis on current and future employment situations (Sinclair et al., 2020). As job insecurity is positively associated with distress and stress, and decreased feelings of meaning in life, it is particularly important for employers to instill feelings of stability among their employees, while being upfront about the impacts of the crisis (Sinclair et al., 2020).

The present findings also provide insight into the significance of promoting feelings of self-efficacy in controlling the virus, as feeling vulnerable to contracting the virus and lack of confidence in preventing the virus were associated with increased stress and distress. Van Bavel et al. (2020) used findings from social and behavioral research to recommend strategies for responding to the COVID-19 pandemic. In particular, the researchers suggested that leaders should instill a sense of collective efficacy among individuals to build trust and compliance (Van Bavel et al., 2020). In the context of COVID-19, building trust is especially important for ensuring socially responsible behavior.

The present findings may also be used to advocate for interventions targeting family functioning, an important resilience factor identified in this study. Many parents are currently facing strain and uncertainty, which is affecting their ability to provide nurturance, guidance, and protection for their children (Walsh, 2015; Prime et al., 2020). As research indicates that adjustment among parents predicts adjustment among children, such disturbances within child rearing may have long-lasting effects on both parents and their children (Hafstad et al., 2010; United Nations, 2020; Wade et al., 2020). These effects can be negated by ensuring parents are provided with proper resources (e.g., peer support, access to pediatricians, financial aid for childcare). This may include online parenting resources (Prime et al., 2020) that help working parents learn techniques (e.g., mindfulness) to cope with work stressors and uncertainty during the COVID-19 crisis (Coyne et al., 2020).

Finally, as social participation was found to be positively associated with one’s meaning in life, it is important to consider how this may be implemented in a time of social distancing. Given that social distancing policies are still in effect, social participation may be difficult to achieve and maintain. Organizations that are primarily maintained through volunteer participation may be additionally affected as some operations may not be able to continue functioning without volunteers. Finding alternative ways (e.g., remote volunteering, social distancing) to conduct essential services, may not only aid organizations but also improve workers’ meaning in life as previously discussed. In line with the importance of social participation, governments in Canada have created online platforms to match interested people with volunteering opportunities.

The current findings demonstrate the need for preventative measures and interventions that utilize a socio-ecological approach. This approach should emphasize the importance of enhancing collaboration between multiple public health and mental health stakeholders to effectively reduce multi-level risk factors present within a global pandemic and to promote resilience factors to improve mental health and wellbeing.

## Concluding Remark

In conclusion, the present study explored the associations of several risk and resilience factors with mental health and wellbeing, and whether resilience factors could buffer the associations of these risk factors with negative outcomes. The current findings highlight the intricate interplay between a vast array of risk and resilience factors that seem to influence workers’ levels of wellbeing and mental health approximately one to two weeks after the implementation of preventative policies, such as social distancing. As the preventative policies in Canada became stricter over the months following these initial weeks before being gradually attenuated during the summer, the impacts of such factors may have also evolved. Thus, future longitudinal research is needed to assess the impact of risk and resilience factors over time, and their continuous and likely cumulative impact on workers’ mental health and wellbeing.

## Data Availability Statement

The raw data supporting the conclusions of this article will be made available by the authors, without undue reservation, to any qualified researcher.

## Ethics Statement

The studies involving human participants were reviewed and approved by Wilfrid Laurier University’s Research Ethics Board. The participants provided their written informed consent to participate in this study.

## Author Contributions

SC oversaw the study and contributed to all steps of the research process, and ran the analysis and contributed to writing the manuscript. TP was the main research coordinator of the study and contributed to the literature review and to writing the manuscript. EC conducted the literature review, and wrote several sections of the manuscript in partnership with CK. MD, EA, and SM were involved in selecting and reviewing the survey measures, and provided their input on the analysis and findings of this manuscript. All authors contributed to the article and approved the submitted version.

## Conflict of Interest

The authors declare that the research was conducted in the absence of any commercial or financial relationships that could be construed as a potential conflict of interest.
